# A C 3 "Bogey"

**Published:** 1921-04-16

**Authors:** 


### The Hospital

The Workers' Newspaper of Administrative Medicine and Institutional Life, Administration, National Insurance and Health.

No. 1819, Vol. LXX. SATURDAY, APRIL 16, 1921. PRICE TWOPENCE

### A C 3 "BOGEY."

The writer of the letter which appeared in *The Times* a few days ago condemns temperately but pointedly the unbridled fury with which the medical correspondent of that newspaper continues to attack the Ministry of Health and all its works. This anonymous assailant has, in fact, overreached himself by the violence of his criticisms. For some mysterious reason he hates the Ministry; and the name of Dr. Addison, who, as a Minister without portfolio, can no longer do him very much harm, still causes him to see red. We hold no brief for the Department over which Dr. Addison lately presided, and we have frequently discussed in a critical spirit many details of its administration; but we prefer to be on the side of fair play, and this perpetual and splenetic disparagement of a great new State service faced with difficult problems of profound importance to the national well-being is not only opposed to the ordinary canons of fair play, but is contrary to the facts and against the best interests of the community.

We have referred in a previous issue to the "various good works" which, as the writer of *The Times* letter reminds the editor, "have been maintained and extended by the Ministry of Health at amazingly little expenditure of money, but with intelligent foresight and unflagging energy." What is of immediate interest is the enthusiasm of *The Times* over the "new official health policy," which it proclaims to be imminent. That policy, it appears, is to be based on the prompt dismissal of the Ministry's "famous bogey, the C 3 nation." There is to be no more "State interference," and all that the new broom is to do is to make a clean sweep of Departmental expenditure. We doubt very much whether this intention is justly attributed to Sir Alfred Mond; we doubt whether he could pursue such a policy of negative futility if he desired to do so. *The Times* may regard with sublime complacency a state of affairs which allows the existence of a million children unfitted to take part in normal school life; it may continue happy in the belief that the problems of tuberculosis, venereal disease, the care of infant life and of the adult worker, and the far-reaching perils of overcrowding will solve themselves if left alone. But a Ministry of Health which regarded any one of these questions as a "bogey" would deserve its fate. It is refreshing to observe the trenchant language recently used by the President of the Medico-Political Union concerning the threatened "squandermania attack against health." If such an attack were to succeed the nation would pay a bitter price.

